# Abnormalities on Chest Computed Tomography and Lung Function Following an Intense Dust Exposure: A 17-Year Longitudinal Study

**DOI:** 10.3390/ijerph16091655

**Published:** 2019-05-13

**Authors:** Charles Liu, Barbara Putman, Ankura Singh, Rachel Zeig-Owens, Charles B. Hall, Theresa Schwartz, Mayris P. Webber, Hillel W. Cohen, Melissa J. Fazzari, David J. Prezant, Michael D. Weiden

**Affiliations:** 1 Pulmonary, Critical Care and Sleep Medicine Division, Department of Medicine and Department of Environmental Medicine, New York University School of Medicine, New York, NY 10016, USA; Charles.Liu@nyulangone.org (C.L.); Barbara.Putman@nyulangone.org (B.P.); 2 The Bureau of Health Services and the FDNY World Trade Center Health Program, Fire Department of the City of New York, Brooklyn, NY 11201, USA; Ankura.Singh@fdny.nyc.gov (A.S.); Rachel.Zeig-Owens@fdny.nyc.gov (R.Z.-O.); Theresa.Schwartz@fdny.nyc.gov (T.S.); Mayris.Webber@fdny.nyc.gov (M.P.W.); David.Prezant@fdny.nyc.gov (D.J.P.); 3 Department of Bioanalysis, Faculty of Pharmaceutical Sciences, Ghent University, 9000 Ghent, Belgium; 4 Pulmonary Medicine Division, Department of Medicine, Montefiore Medical Center and Albert Einstein College of Medicine, Bronx, NY 10467, USA; 5 Division of Epidemiology, Department of Epidemiology and Population Health, Albert Einstein College of Medicine, Bronx, NY 10461, USA; Hillel.Cohen@einstein.yu.edu; 6 Division of Biostatistics, Department of Epidemiology and Population Health, Albert Einstein College of Medicine, Bronx, NY 10461, USA; Charles.Hall@einstein.yu.edu (C.B.H.); Melissa.Fazzari@einstein.yu.edu (M.J.F.)

**Keywords:** medical imaging, pulmonary function tests, lung injury, occupational exposure, epidemiological studies

## Abstract

Fire Department of the City of New York (FDNY) firefighters experienced intense dust exposure working at the World Trade Center (WTC) site on and after 11/9/2001 (9/11). We hypothesized that high-intensity WTC exposure caused abnormalities found on chest computed tomography (CT). Between 11/9/2001–10/9/2018, 4277 firefighters underwent a clinically-indicated chest CT. Spirometric measurements and symptoms were recorded during routine medical examinations. High-intensity exposure, defined as initial arrival at the WTC on the morning of 9/11, increased the risk of bronchial wall thickening, emphysema, and air trapping. Early post-9/11 symptoms of wheeze and shortness of breath were associated with bronchial wall thickening, emphysema, and air trapping. The risk of accelerated forced expiratory volume at one second (FEV_1_) decline (>64 mL/year decline) increased with bronchial wall thickening and emphysema, but decreased with air trapping. The risk of airflow obstruction also increased with bronchial wall thickening and emphysema but decreased with air trapping. In a previously healthy occupational cohort, high-intensity WTC exposure increased the risk for CT abnormalities. Bronchial wall thickening and emphysema were associated with respiratory symptoms, accelerated FEV_1_ decline, and airflow obstruction. Air trapping was associated with respiratory symptoms, although lung function was preserved. Physiologic differences between CT abnormalities suggest that distinct types of airway injury may result from a common exposure.

## 1. Introduction

The collapse of the World Trade Center (WTC) on the morning of 11 September 2001 (9/11) produced a caustic dust plume containing more than 10,000,000 tons of irritating alkaline dust with pH > 10 [1]. Fire Department of the City of New York (FDNY) rescue and recovery workers who arrived at the site the morning of 9/11, were exposed to an extremely high concentration of dust that produced airway injury. Those who arrived in the afternoon and following days received a lower intensity, but substantial exposure, as rescue and recovery work resuspended the dust. WTC exposure produced an acute drop in forced expiratory volume at one second (FEV_1_) and forced vital capacity (FVC) in FDNY workers present at the site prior to 24/9/2001 [2,3,4]. In the years following 9/11, WTC-exposed rescue/recovery workers had high rates of airway injury, including excessive loss of lung function [2], airflow obstruction [3], and airway hyper-reactivity [4]. A significant portion of the cohort experienced accelerated FEV_1_ decline, defined as greater than 64 mL/year FEV_1_ decline, a risk factor for chronic obstructive pulmonary disease (COPD) [5] and asthma/COPD overlap [6].

Computed tomography (CT) can detect structural lung abnormalities in individuals with respiratory symptoms and normal spirometry [7]. Self-report of exposure to vapors, gas, dust, or fumes was associated with an increase in emphysema, bronchial wall thickening, and air trapping on quantitative CT imaging in cohorts of smokers without WTC exposure [8,9]. Bronchial wall thickening was associated with FEV_1_ decline in WTC-exposed cohorts [3,10,11]. Air trapping on expiratory CT imaging is a manifestation of small airway disease [12,13] and has been observed in a number of WTC-exposed cohorts [10,14]. The relationship between WTC dust exposure and these CT abnormalities, however, is still poorly defined.

The aim of this study was to determine predictors of WTC-related airway CT abnormalities on clinically-indicated chest CT (*N* = 4277). The main predictor of interest was high-intensity WTC dust exposure, defined as initial arrival at the WTC site during the morning of 9/11. We also examined the associations of emphysema, bronchial wall thickening and air trapping with early respiratory symptoms and longitudinal lung function in order to understand the clinical correlates of radiographic airway abnormalities in this population.

## 2. Methods

### 2.1. Study Population

**Figure 1:**
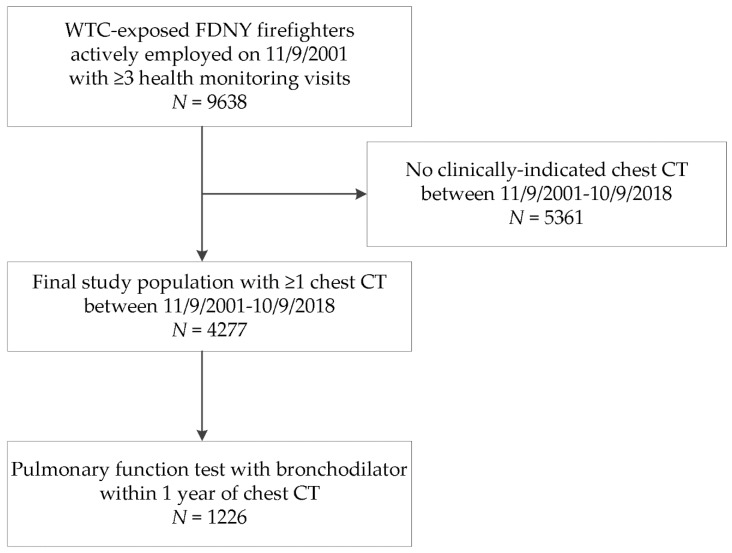
Firefighters who participated in the chest computed tomography (CT) study. Shown is the source population of male firefighters who were employed by the Fire Department of the City of New York (FDNY) on 11/9/2001, present at the World Trade Center (WTC) site between 11/9/2001 and 24/9/2001, and had at least three monitoring pulmonary function tests (PFTs) between 11/9/2001–10/9/2018 to assess longitudinal forced expiratory volume at one second (FEV_1_) and FEV_1_/FVC (forced vital capacity). The final study population included those who had received a clinically-indicated chest CT scan between 11/9/2001 and 10/9/2018. A subgroup had a clinically-indicated bronchodilator PFT within one year of the chest CT.

The source population consisted of 9638 male firefighters who were actively employed by the FDNY on 9/11, first arrived at the WTC site between 11/9–24/9/2001, and had ≥3 post-9/11 routine medical monitoring spirometries taken at FDNY [5]. A subset of this population received at least one clinically-indicated chest CT at a hospital-based radiology facility between 11/9/2001 and 10/9/2018. The final study population included 4277 firefighters (Figure 1). Participants provided written informed consent. The Montefiore Medical Center (FWA #00002558)/Albert Einstein College of Medicine (FWA #00023382) Institutional Review Board approved this study.

### 2.2. CT Scans

Inspiratory and expiratory noncontrast chest CTs were performed at a hospital-based radiology facility. Clinical indications for chest CT included symptoms or reductions in spirometry. All CT scans received standard radiologic evaluation. Radiologists were unaware of intensity of WTC exposure, and radiologist reports were entered into the FDNY medical record in real-time. The findings of emphysema, bronchial wall thickening, and air trapping were abstracted and entered into the FDNY medical record. The first post-9/11 CT scan report was used for analysis.

### 2.3. Participant Characteristics

Demographic data (age and race) were obtained from the FDNY employee database. Participants’ height, weight, and self-reported smoking status (ever or never-smoker) were assessed during routine medical monitoring examinations at FDNY, scheduled once every 12–18 months. Those who consistently self-reported no cigarette smoking were classified as never-smokers. Those who ever reported current or former smoking on medical monitoring exams were defined as ever-smokers.

### 2.4. World Trade Center Dust Exposure

Participants’ time of initial arrival at the WTC site was determined during the first medical monitoring examination. We classified individuals who arrived at the site on the morning of 9/11 as having high-intensity WTC exposure [4].

### 2.5. Longitudinal Respiratory Symptoms

A total of 44,832 medical monitoring exams, which included administration of health questionnaires and spirometry, were performed in the study population between 2/10/2001 and 10/9/2018 (median per individual: 11, interquartile range: 9–12). Reports of lower respiratory symptoms were collected via the self-administered physical health questionnaires [15]. For the respiratory symptoms within 6 months post 9/11, the first monitoring exam was used.

### 2.6. Longitudinal Screening Spirometry

FEV_1_ and FVC measurements were obtained from spirometry performed during the monitoring examinations. Spirometry was carried out as described in our previous studies [2,4]. Post-9/11 rates of FEV_1_ decline (FEV_1_ slopes) were estimated for each participant using the first post-9/11 FEV_1_ and all subsequent FEV_1_ measurements in linear regression analyses that modeled FEV_1_ as a linear function of time since 9/11. An FEV_1_ slope of more than 64 mL/year was defined as accelerated FEV_1_ decline. Airflow obstruction was defined as two consecutive FEV_1_/FVC ratios less than 0.70; we required that measurements be at least one year apart [5].

### 2.7. Complete Pulmonary Function Testing

A subset of participants underwent complete pulmonary function tests (PFTs), which were performed according to American Thoracic Society standards [16] at a hospital-based pulmonary function laboratory. Because the Global Initiative for Chronic Obstructive Lung Disease definition of airflow limitation requires a postbronchodilator FEV_1_/FVC ratio < 0.70, we conducted a sensitivity analysis within the participants who had had a complete PFT, which included pre- and postbronchodilator flow rates [17]. Total lung capacity, functional residual capacity (FRC) and residual volume (RV) measurements were also available from the complete PFT data; these were measured prior to bronchodilator administration and were used to calculate expiratory reserve volume (FRC minus RV).

### 2.8. Statistical Analyses

Demographic and other characteristics of the study population were assessed as *n* (%) and means (±SD). Chi-square tests were used to evaluate associations between bronchial wall-thickening, emphysema, and air-trapping. Hierarchic cluster analysis of the CT abnormalities was performed and a dendrogram was generated using SAS proc varclus with centroid method. We performed log-rank Mantel–Cox tests to examine the univariable associations of WTC exposure with the CT diagnoses of air trapping, emphysema, and bronchial wall thickening. Three separate multivariable Cox regression models assessed the associations of WTC exposure intensity with the outcomes emphysema, bronchial wall thickening, and air trapping. These analyses were repeated in the subset of the cohort that underwent medical monitoring in the first six months after 9/11 in order to investigate the relationship between early post-9/11 respiratory symptoms and abnormalities on chest CT (*N* = 3610). Follow-up time for these Cox models started at 9/11 and ended at the date of the CT scan. Parametric interval-censored survival models were used as sensitivity analyses, as the date of incidence of abnormalities would necessarily be prior to the CT on which they are identified. As a secondary analysis we performed a logistic regression to estimate associations between CT abnormalities and respiratory symptoms throughout the follow-up period, adjusting confidence intervals and *p*-values for potential overdispersion using the Pearson goodness-of-fit statistic. We then used logistic regression models to estimate associations of CT abnormalities with accelerated FEV_1_ decline and airflow obstruction. We first used minimally-adjusted models, with age on 9/11 and race as the only covariates, and later added the covariates smoking status at the end of follow-up, first post-9/11 FEV_1_, first post-9/11 BMI, and WTC exposure level. Covariates were selected based on previously known factors that affect lung physiology.

Linear mixed models with random intercepts were used to examine longitudinal FEV_1_ % predicted and FEV_1_/FVC ratio trajectories in those who had CT abnormalities. Participants’ age on 9/11, height, and race were included as fixed effects in the models with FEV_1_/FVC ratio as the outcome. Mean FEV_1_ % predicted and FEV_1_/FVC ratio were estimated for each 1-year period between 11/9/2000 and 10/9/2018 for four separate groups based on the following CT outcomes: no CT diagnosis, isolated air trapping, isolated emphysema, and isolated bronchial wall thickening. For these analyses, we excluded 403 participants with more than one CT abnormality.

Two analyses were performed in the subset of study participants with complete PFTs (*N* = 1226). A multivariable logistic regression model assessed the associations between the CT abnormalities and airflow obstruction (post-bronchodilator FEV_1_/FVC ratio < 0.70), and multivariable linear regression models were used to assess the associations between CT abnormalities and prebronchodilator lung volumes. Age on 9/11, race, BMI, smoking status at the end of follow-up, WTC exposure level and first post-9/11 FEV_1_ were included as covariates. As stated above, covariates were selected based on previously known factors that affect lung physiology.

Reported *p*-values are two-sided and considered significant at the < 0.05 level. Data analyses and dendrogram were performed using SAS version 9.4 (SAS Institute, Inc., Cary, NC, USA). Cumulative incidence curves and lung function figures were created with Prism 8 (GraphPad Software, Inc., San Diego, CA, USA).

## 3. Results

### 3.1. Population Characteristics

The final study population, with clinically-indicated inspiratory and expiratory chest CT, was 37% of the source population. The CT scans were performed throughout the entire follow-up period, with a peak at year six post-9/11; this was due to increased outreach to recently retired WTC-exposed firefighters at that time (Figure S1 in Supplementary Materials). Demographic and other characteristics of the 4277 firefighters in the final study population (Figure 1) and those without chest CT scans are presented in Table 1. Fewer than 1% of study participants had missing covariate data. Compared with WTC-exposed firefighters who did not have a clinically indicated chest CT, the study population with a clinically indicated chest CT was slightly different in that it was older, had a greater proportion of ever-smokers, higher intensity WTC exposure (arrival at the WTC site on the morning of 9/11), lower postexposure lung function, and had a higher proportion of reports of shortness of breath and wheeze within six months of 9/11.

### 3.2. CT Abnormalities and WTC Exposure

**Figure 2:**
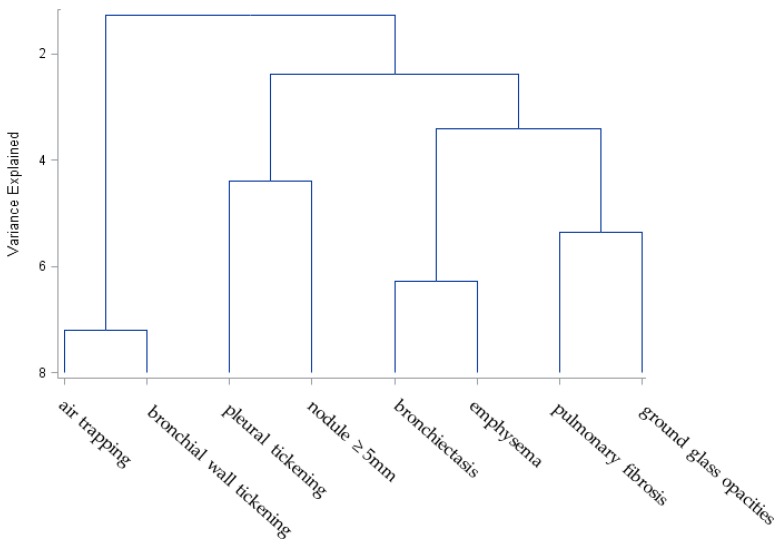
Chest CT abnormalities in WTC-exposed firefighters. The dendrogram demonstrates clustering of abnormalities on chest CTs.

Inspiratory and expiratory chest CTs diagnosed bronchial wall thickening in 837 individuals (20%) and air trapping in 894 (21%). Nodules greater than or equal to 5 mm and ground glass opacities were less common but present in over 10% of participants (Table 2). Emphysema, pleural thickening, and bronchiectasis were present in between 3% and 6% of participants. Pulmonary fibrosis was rare, present in just 0.6% of individuals with a CT. Bronchial wall thickening was correlated with air trapping and emphysema (*p* < 0.001 and *p* < 0.001, respectively), but air trapping and emphysema were not correlated with one another (*p* = 0.72). Hierarchical clustering demonstrated that bronchial wall thickening clustered with air trapping (Figure 2). Bronchiectasis and emphysema clustered but were distantly related to bronchial wall thickening and air trapping. The parenchymal abnormalities of pleural thickening and pulmonary nodules clustered as did ground glass opacities and pulmonary fibrosis.

**Figure 3:**
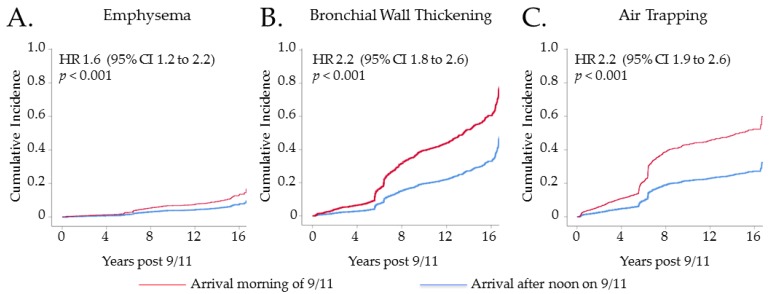
Cumulative incidence of chest CT diagnosis according to WTC exposure intensity. (**A**) shows the cumulative incidence of emphysema in participants who arrived morning of 9/11 at the WTC site (red line) compared with those who arrived between the afternoon of 9/11 and 24/9/2001 (blue line). (**B**) shows the cumulative incidence of bronchial wall thickening in those who arrived morning of 9/11 (red) and those who arrived later (blue). (**C**) shows the cumulative incidence of air trapping in participants who arrived morning of 9/11 (red) and those who arrived later (blue).

Univariable analyses showed that high-intensity WTC exposure is associated with a 2.2-fold increased risk of air trapping, a 1.6-fold increased risk of emphysema, and a 2.2-fold increase in the risk of bronchial wall thickening Figure 3. The associations between high-intensity WTC exposure and chest CT diagnoses persisted in a multivariable Cox regression model (Table 3). Emphysema was strongly associated with ever-smoking. Bronchial wall thickening was also associated with ever-smoking, though the magnitude of association was lower.

### 3.3. CT Abnormalities and Respiratory Symptoms

Respiratory symptoms of shortness of breath and wheezing were common during the first six months after 9/11; 28.2% (1,018/3,610) reported a single symptom of either shortness of breath or wheezing, and 17.5% (630/3,610) reported both shortness of breath and wheezing. Early respiratory symptoms increased the risk of air trapping, emphysema, and bronchial wall thickening according to a Cox model adjusted for age, race, BMI, smoking status, WTC exposure and first post-9/11 FEV_1_. Having both shortness of breath and wheezing was a greater risk factor for each of the CT abnormalities than having either symptom by itself (Table 4). We also assessed persistence of symptoms in longitudinal follow-up. Participants with air trapping, emphysema, or bronchial wall thickening on chest CT reported shortness of breath and/or wheeze more frequently during medical monitoring exams between 11/9/2001 and 10/9/2018 than those without one of those abnormalities on chest CT. (data not shown).

### 3.4. CT Abnormalities and Longitudinal Spirometry

To assess spirometric correlates of CT abnormalities, we tested the associations of emphysema, bronchial wall thickening, and air trapping with accelerated FEV_1_ decline. A minimally-adjusted logistic regression model, adjusted for only age and race, showed similar results as Table 5 (data not shown). Emphysema and bronchial wall thickening were associated with increased risk of accelerated FEV_1_ decline in a multivariable-adjusted logistic model (Table 5), and air trapping was associated with reduced odds of accelerated FEV_1_ decline.

**Figure 4:**
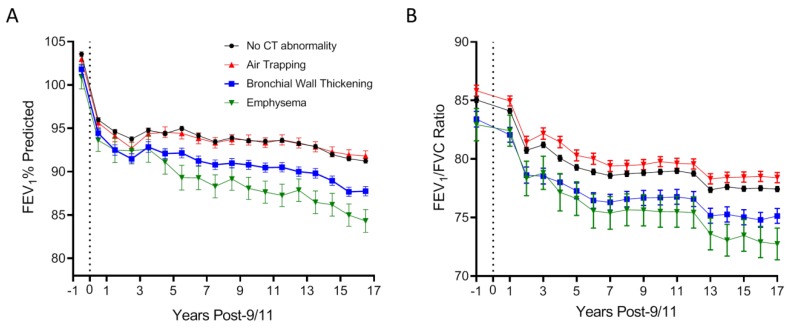
Longitudinal lung function according to CT abnormality. (**A**) shows mean FEV_1_ % predicted (± SEM) in each year between 11/9/2000 and 10/9/2018 in the no chest CT diagnosis (black circles), isolated air trapping (blue triangles), isolated bronchial wall thickening (brown squares), and isolated emphysema (red inverted triangle) groups. (**B**) shows mean (± SEM) FEV_1_/FVC ratio in each year in the aforementioned groups, adjusted for age, race and height. Number of subjects in each nonoverlapping group is shown. The vertical line at 0 represents 11/9/2001.

We then examined how airway abnormalities on CT were associated with longitudinal FEV_1_ and FEV_1_/FVC ratio using stratified mixed effect linear models (Figure 4). Isolated emphysema was associated with the most rapid longitudinal FEV_1_ decline, while FEV_1_ decline in those with isolated bronchial wall thickening was intermediate between the isolated emphysema and air trapping subgroups (Figure 4A).

A different pattern emerged when examining longitudinal FEV_1_/FVC ratio (Figure 4B). When compared with individuals with no airway abnormalities on CT, isolated air trapping was associated with increased FEV_1_/FVC ratio both before and after 9/11. FEV_1_/ FVC ratio was lowest in the isolated emphysema subgroup, and intermediate in those with isolated bronchial wall thickening.

We then tested whether having airway abnormalities on CT was associated with airflow obstruction, defined as either two consecutive FEV_1_/FVC ratios < 0.70 on screening spirometry (*N* = 4277) or as postbronchodilator FEV_1_/FVC ratio <0.70 in the subgroup with complete PFTs (*N* = 1226). Minimally-adjusted logistic regression, adjusting for age and race only, showed that both emphysema and bronchial wall thickening increased the odds of airflow obstruction (OR: 3.34, 95% CI: 2.47–4.51 and OR: 2.19, 95% CI: 1.04–1.07), but air trapping was associated with a reduced odds of this outcome (OR: 0.46, 95% CI: 0.34–0.62). Emphysema and bronchial wall thickening were also associated with an elevated odds of airflow obstruction in multivariable logistic regression analyses that included age, race, BMI, smoking status, first post-9/11 FEV_1_, and WTC exposure level (Table 6). Air rapping was still associated with a lower odds of airflow obstruction after adjusting for these additional covariates. Analyses performed in the subpopulation with complete PFTs showed similar results.

### 3.5. CT Abnormalities and Lung Volumes

We evaluated the associations of emphysema, bronchial wall thickening, and air trapping with lung volumes in the subgroup with complete PFTs. In multivariable linear regression analyses, emphysema, bronchial wall thickening, and air trapping were associated with greater total lung capacity. Emphysema and bronchial wall thickening were also associated with increased functional residual capacity, while air trapping was associated with reduced expiratory reserve volume (Table 7).

## 4. Discussion

This study documented that firefighters who arrived at the WTC site on the morning of 9/11 (high-intensity exposure) had a higher risk of subsequent airway abnormalities on chest CT than those with lesser WTC exposure. This supports the hypothesis that WTC exposure contributed to the development of these airway abnormalities. Those with the highest dust exposure had an increased odds of subsequent emphysema, bronchial wall thickening and air trapping after controlling for potential confounders. The increased risk of CT abnormalities following high-intensity WTC exposure suggests that a massive but brief irritant contact is a risk factor for radiographic abnormalities even years after the exposure. Because both the high and low WTC exposure groups share the non-WTC occupational exposure of firefighting, the increased risk of CT abnormalities in the high exposure group is therefore attributable to WTC exposure. These data are consistent with prior case-control studies in WTC-exposed cohorts [10] and in occupational cohorts with high smoking prevalence [8,9].

The airway abnormalities on CT were associated with early respiratory symptoms in this cohort. Large cohort studies in the general population have demonstrated associations between respiratory symptoms and CT abnormalities including emphysema, bronchial wall thickening, and air trapping [7,18,19,20]. In our population, those with CT abnormalities had elevated functional residual capacity. Similar elevated lung volumes have been observed in other WTC cohorts [14], suggesting that dynamic hyperinflation could contribute to respiratory symptoms even in individuals with normal FEV_1_ and FEV_1_/FVC ratio [21].

Unlike other dust-exposed occupational cohorts, WTC exposed firefighters were a health occupational cohort prior to WTC exposure with normal lung function [2]. This could be a reason why certain CT abnormalities were rare. Pulmonary fibrosis was present in only 0.6% of the first post-9/11 clinically indicated chest CTs. Similarly, ground glass opacities, pulmonary nodules, and pleural thickening were uncommon present in only 3 to 15%.

Only 6% of the study population had emphysema, likely due to the healthy worker effect of a physically demanding occupation, and the fact that there were fewer smokers than in the general population. As expected, emphysema was strongly associated with cigarette smoking, accelerated FEV_1_ decline, and airflow obstruction. Importantly, those with the most intense dust exposure had an increased risk of emphysema when compared with those with less intense exposure after adjusting for potential confounders. This suggests that those caught in the dust cloud on the morning of 9/11, especially ever-smokers, will continue to be at a higher risk for developing emphysema than those with less intense WTC dust exposure.

While bronchial wall thickening and air trapping cluster closely together, air trapping is a physiologically distinct manifestation of dust-induced small airway injury. Unlike bronchial wall thickening, small airways disease leading to air trapping was associated with reduced odds of airflow obstruction in this cohort. Reduction of expiratory reserve volume associated with air trapping likely contributes to the observed reduction of FVC. This could account for the elevated longitudinal FEV_1_/FVC ratio. The preservation of FEV_1_ in those with air trapping suggests the disease process spares the larger airways that limit forced expiratory airflow. In our cohort, the impact of high-intensity exposure on subsequent air trapping was stronger in never-smokers than ever-smokers. In non-WTC cohorts, excessive FEV_1_ decline was observed in those with smoking-associated and age-related air trapping [12,13,22]. Furthermore, in smokers, air trapping may be a transition state to emphysema [23]. In our cohort with preserved lung function and low smoking prevalence, air trapping was not associated with smoking or emphysema, suggesting that the mechanism of small airway injury or stage of disease may influence the relationships between CT abnormalities and FEV_1_ decline. This highlights the need to use different measures of distal airway function, such as impulse oscillometry, to assess and follow the physiology of small airway abnormalities associated with air trapping on CT [24,25,26]. Further research is needed to define effective treatments in those who have air trapping-associated symptoms, but preserved FEV_1_ and FEV_1_/FVC ratio.

Firefighters with bronchial wall thickening had greater longitudinal FEV_1_ decline and airflow obstruction when compared with those having no airway abnormalities or air trapping. Bronchial wall thickening had a phenotype that was similar to but less severe than emphysema, with increased total lung capacity, functional residual capacity, and increased risk of accelerated FEV_1_ decline and airflow obstruction. Greater FEV_1_ decline observed on serial spirometry is a strong risk factor for airflow obstruction [5]. The increased risk of airflow obstruction associated with bronchial wall thickening was similar regardless of whether obstruction was determined by FEV_1_/FVC < 0.70 in the entire study group (serial spirometry) or in the subgroup with postbronchodilator measurements. This suggests that misclassification or selection bias associated with the different definitions of obstruction did not change the associations with CT abnormalities. The association of bronchial wall thickening with accelerated FEV_1_ decline highlights the utility of CT in risk assessment of symptomatic patients with preserved lung function [7]. Future investigation of longitudinal CT scans is needed to assess if bronchial wall thickening is a risk factor for developing emphysema, especially in never-smokers.

There are several limitations to this investigation. FDNY firefighters are overwhelmingly white, male, and previously healthy workers, potentially limiting generalizability of these findings; however, most findings from the FDNY cohort have been replicated in other WTC-exposed cohorts [10,11,14]. The reported CT abnormalities were real world data defined by clinical radiologists without quantitative image analysis or inter-reader reliability measures. Nonetheless, there were strong correlations between CT abnormalities and exposure intensity. CT airway abnormalities also correlated with PFT measurements, including lung volumes, suggesting validity of the clinical CT interpretation. Lastly, since the CT scans were clinically indicated, this study was vulnerable to selection bias. The study population differed from those without chest CTs, with more intense WTC exposure, more respiratory symptoms and worse postexposure lung function. This precludes assessment of the incidence of abnormal CTs in the entire cohort, since undiagnosed cases are likely. Nevertheless, analyses within the CT population provide a valid assessment of risk factors for specific diagnoses within a symptomatic subgroup.

## 5. Conclusions

In WTC-exposed firefighters, high-intensity dust exposure was associated with subsequent bronchial wall thickening, air trapping, and emphysema, a causal link between WTC exposure and these CT abnormalities. Emphysema and bronchial wall thickening were associated with airflow obstruction and an accelerated rate of longitudinal FEV_1_ loss. Isolated air trapping identifies a symptomatic subgroup without excessive FEV_1_ decline or airflow obstruction.

The findings from this study are a valuable resource for understanding irritant-induced airways disease in a previously healthy population. In those with radiographic abnormalities, the 17-year decline in lung function is still mild enough that the potential exists for interventions, such as tobacco cessation or pharmacologic targeting of specific inflammatory pathways, to improve lung function trajectory and respiratory symptoms.

## Figures and Tables

**Table 1:** Population characteristics *.

Variable	Population without Chest CT *N* = 5361	Chest CT Study Population *N* = 4277
Age on 9/11	39.4 ± 7.5	41.2 ± 7.2
BMI ^‡^	28.7 ± 3.4	29.0 ± 3.5
Smoking Status, *N* (%)		
Never	3804 (71.0)	2653 (62.0)
Ever	1557 (29.0)	1624 (38.0)
Race, *N* (%)		
White	5026 (93.8)	4053 (94.8)
Black	139 (2.6)	86 (2.0)
Hispanic	180 (3.4)	128 (3.0)
Other	16 (0.3)	10 (0.2)
World Trade Center exposure, *N* (%)		
Morning of 9/11	463 (8.6)	1113 (26.0)
Afternoon on 9/11–9/12	4125 (76.9)	2781 (65.0)
9/13–9/24	773 (14.4)	383 (9.0)
First Post-9/11 Spirometry		
FEV_1_ % predicted	98.3 ± 13.1	95.4 ± 14.2
FVC % predicted	93.4 ± 11.8	91.1± 12.3
FEV_1_/FVC	0.84 ± 0.05	0.83 ± 0.06
Post-9/11: FEV_1_ slope (mL/year)	−34.6 ± 25.6	−38.9 ± 30.5
Report of respiratory symptoms within 6 months of 9/11		
Shortness of breath	1004 (22.3) ^§^	1289 (35.7) ^‖^
Wheeze	797 (17.7) ^§^	989 (27.4) ^‖^

* All values are mean ± standard deviation unless otherwise stated. ^‡^ Body Mass Index. ^§^ *N* = 4500. ^‖^ *N* = 3610.

**Table 2:** Prevalence of CT abnormality.

CT Abnormality	Percentage of Chest CT Scans with Abnormality
Air Trapping	20.9
Bronchial Wall Thickening	19.6
Nodules ≥ 5 mm	14.6
Ground Glass Opacities	12.2
Emphysema	5.9
Bronchiectasis	3.6
Pleural Thickening	3.0
Pulmonary Fibrosis	0.6

Note: abnormalities are not mutually exclusive.

**Table 3:** Cox regression models predicting three Chest CT abnormalities ^a,b^.

Variables	Emphysema			Bronchial Wall Thickening			Air Trapping		
	HR	95% CI	*p*	HR	95% CI	*p*	HR	95% CI	*p*
WTC exposure morning of 9/11	1.83	1.36–2.45	<0.001	2.33	2.00–2.72	<0.001	2.34	1.95–2.80	<0.001
Ever-smoker ^c^	7.04	4.94–10.04	<0.001	1.25	1.08–1.44	0.003	0.73	0.61–0.88	0.001

^a^*N* = 4277. ^b^ Adjusted for age, race, BMI and first post-9/11 FEV_1_. ^c^ Reference is never-smoker.

**Table 4:** Subpopulation with respiratory symptoms reported within six months of exposure ^a,b^.

Variables	Emphysema			Bronchial Wall Thickening			Air Trapping		
	HR	95% CI	*p*	HR	95% CI	*p*	HR	95% CI	*p*
Either shortness of breath or wheeze	1.43	1.04–1.97	0.03	1.38	1.16–1.63	<0.001	1.27	1.08–1.49	0.005
Both shortness of breath and wheeze	1.62	1.12–2.33	0.01	1.49	1.22–1.83	<0.001	1.41	1.16–1.70	<0.001

^a^*N* = 3610. ^b^ Adjusted for age, race, BMI, smoking status, WTC exposure and first post-9/11 FEV_1_.

**Table 5:** Multivariable logistic regression model assessing associations of CT diagnoses with accelerated FEV_1_ decline *^,†^.

CT Diagnosis	OR	95% CI
Emphysema	1.89	1.37–2.60
Bronchial Wall Thickening	1.55	1.25–1.92
Air Trapping	0.77	0.61–0.97

* N = 4277; ^†^ Adjusted for age, race, BMI, smoking status, WTC exposure and first post 9/11 FEV_1_.

**Table 6:** Multivariable logistic regression models assessing association of CT diagnosis with two definitions of airflow obstruction*.

CT Diagnosis	Two Consecutive Screening Spirometric FEV_1_/FVC < 0.70 N = 4277		Post-BD ^$^ FEV_1_/FVC < 0.70 N = 1226	
	OR	95% CI	OR	95% CI
Emphysema	2.03	1.44–2.88	2.63	1.56–4.42
Bronchial Wall Thickening	2.25	1.77–2.87	2.67	1.84–3.88
Air Trapping	0.40	0.29–0.55	0.36	0.22–0.58

* Adjusted for age, race, BMI, smoking status, WTC exposure and first post-9/11 FEV_1_. ^$^ Post-bronchodilator.

**Table 7:** Multivariable linear models assessing the association of CT abnormalities with lung volumes ^a,b^.

CT Abnormality	Total Lung Capacity ^c^			Functional Residual Capacity ^c^			Expiratory Reserve Volume ^c^		
	Beta	95% CI	*p*	Beta	95% CI	*p*	Beta	95% CI	*p*
Air Trapping	57	−76, 189	0.40	−71	−169, 27	0.16	−205	−281, −129	<0.001
Emphysema	571	357, 786	<0.001	478	319, 637	<0.001	161	38, 285	0.01
Bronchial Wall Thickening	300	169, 432	<0.001	236	139, 334	<0.001	−46	−122, 30	0.23

^a^ Adjusted for age, race, BMI, smoking status, WTC exposure and first post-9/11 FEV_1_. ^b^ mL. ^c^ *N* = 1226 due to missing covariates. ^d^ *N* = 1224 due to missing covariates.

## Supplementary Materials

The following are available online at https://www.mdpi.com/1660-4601/16/9/1655/s1, Figure S1: Distribution of the timing of chest CT. The histogram is showing the number of chest CT scans used for this study, grouped by year post-9/11. There is a peak at year 6–7 post-9/11, with 1049/4277 (25%) CT scans performed that year.
