# Supplementary material for: Abnormalities on Chest Computed Tomography and Lung Function Following an Intense Dust Exposure: A 17-Year Longitudinal Study
Source: Int J Environ Res Public Health. 2019 May 13;16(9):1655. doi: 10.3390/ijerph16091655 (PMC6540073; doi:10.3390/ijerph16091655)
Supplement: Supplementary file 1 [file ijerph-16-01655-s001.pdf]

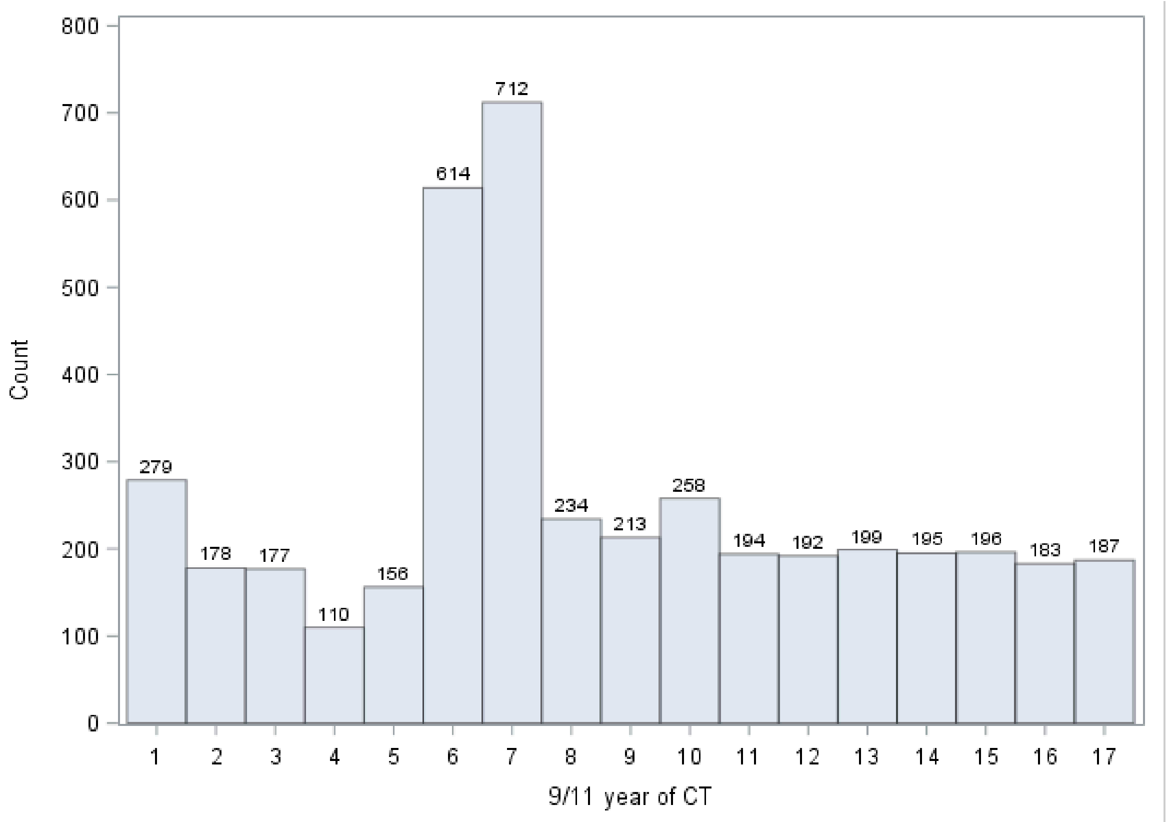

Figure S1: Distribution of the timing of chest CT. The histogram is showing the number of chest CT scans used for this study, grouped by year post-9/11. There is a peak at year 6–7 post-9/11, with 1049/4277 (25%) CT scans performed that year.
